# Application of *Aloe vera* Gel Coating Enriched with Cinnamon and Rosehip Oils to Maintain Quality and Extend Shelf Life of Pomegranate Arils

**DOI:** 10.3390/foods11162497

**Published:** 2022-08-18

**Authors:** Jagmeet Singh, Sunil Pareek, Vaibhav Kumar Maurya, Narashans Alok Sagar, Yogesh Kumar, Prarabdh C. Badgujar, Olaniyi Amos Fawole

**Affiliations:** 1 Department of Agriculture and Environmental Sciences, National Institute of Food Technology Entrepreneurship and Management, Kundli, Sonepat 131 028, Haryana, India; 2 Department of Basic and Applied Sciences, National Institute of Food Technology Entrepreneurship and Management, Kundli, Sonepat 131 028, Haryana, India; 3 Department of Food Science and Technology, National Institute of Food Technology Entrepreneurship and Management, Kundli, Sonepat 131 028, Haryana, India or or; 4 Postharvest Research Laboratory, Department of Botany and Plant Biotechnology, University of Johannesburg, Johannesburg 2006, South Africa

**Keywords:** *Aloe vera*, edible coating, essential oil, organoleptic attributes, phenolics, pomegranate arils, quality attributes, rosehip oil, storage

## Abstract

A completely randomized design was applied on pomegranate arils for several post-harvest treatments before the packaging in polypropylene boxes for 15 days at (5 ± 1 °C, 95 ± 2% RH): control (untreated), *Aloe vera* gel (10% or 20%), 10% *Aloe vera* + rosehip oil (0.25% or 0.50%), 20% *Aloe vera* + rosehip oil (0.25% or 0.50%), 10% *Aloe vera* + cinnamon oil (0.25% or 0.50%), and 20% *Aloe vera* + cinnamon oil (0.25% or 0.50%). *Aloe vera* in combination with cinnamon oil resulted in an enhanced shelf life (15 d) compared to the uncoated arils (control). The *Aloe vera* + cinnamon oil coating led to the retention of total phenolics, anthocyanin, ascorbic acid, and antioxidant activity in context to the quality attributes. Moreover, this coating showed minimal change in the color, total soluble solids, titratable acidity, firmness, delayed ethylene production, respiration rate, and physiological weight loss. Also, *A. vera* + cinnamon oil coatings significantly (*p *< 0.05) inhibited the total counts of mesophilic aerobics, coliforms, and yeast and mold. Organoleptic attributes, including color, flavor, aroma, texture, and purchase acceptability were higher for the arils that were treated with 10% *A. vera* + 0.25% cinnamon oil; thus, this highly economical and easily available coating material can be formulated and used commercially to extend the shelf life and enhance the profit of the producers and/or processors.

## 1. Introduction

Pomegranate (*Punica granatum* L.) fruit is one of the oldest edible fruit that is cultivated extensively in Spain, Egypt, Russia, India, France, China, Japan, the USA, South Africa, and Iran. The edible part of fruit consists of arils, which constitute 52% of the total fruit weight and contain 78% (*w*/*w*) juice and 22% (*w*/*w*) seeds [1]. There has been an extraordinary increase in consumer interest in pomegranate in the past decades due to the high-quality attributes, unique flavors and taste, antioxidant properties of arils, and their potential health advantages [2,3].

Although pomegranate is non-climacteric, the fruit is characterized by accelerated quality loss resulting in reduced shelf life. The arils are consumed fresh or as processed juice-based products. Minimally processed ‘ready-to-eat’ pomegranate arils in modified atmospheric (MA) packaging have become very popular due to their convenience, sensory attributes, and health benefits [4]. However, maintaining the nutritional and microbial quality of pomegranate arils is a major challenge since minimally processed arils easily lose quality characteristics such as texture and color, together with increased microbial spoilage [5,6]. Only a 10-day shelf life was observed in MA-packaged arils, and it was limited to only 7-days if the flavor and aroma were taken into account [7].

Thus, new alternatives are required to reduce the microbial population on pomegranate arils and delay quality loss. Edible coatings have emerged as alternatives to preserve fresh and minimally processed commodities, as they provide a partial barrier to moisture, oxygen, and carbon dioxide, thereby improving the mechanical handling properties, carrying additives, avoiding the loss of volatiles, and sometimes contributing to the production of aroma volatiles [8].

Nevertheless, few studies have dealt with the preservation of pomegranate arils using various edible coatings, mainly chitosan coating. Chitosan and ascorbic acid coating retained the visual quality of the pomegranate arils during storage and inhibited bacterial and fungal growth on them [9]. However, chitosan is considered a non-vegetarian origin and, therefore, has an acceptance issue in the vegetarian population. As a result, *Aloe vera* (AV) gel has gained much attention for use as a safe and environmentally friendly post-harvest coating. Previous reports have shown that AV gel can be used in preserving several fruit commodities, including apple [10], grapes [11], kiwifruit [12], mango [13], nectarine [14], papaya [15], peach [16], plum [17], pomegranate arils [18], sweet or sour cherry [19], and some stone fruits [20]. The coating based on AV effectively reduced respiration rate, weight loss, color changes, and the total acidity, and ethylene production in climacteric fruits, resulting in quality maintenance and shelf life extension of the treated fruits.

AV gel consists of polysaccharides followed by soluble sugars, proteins, vitamins, and minerals, while the lipid content is very low, ranging between 0.07 and 0.42% depending on *Aloe* spp. [21]. Martinez-Romero et al. [18] investigated the effect of AV gel alone or in combination with ascorbic and citric acids on the overall quality of minimally processed pomegranate arils during storage under MA-packaging conditions. Although the AV + citric acid application maintained the quality parameters and reduced microbial spoilage of the arils, the shelf life was limited to 12 days [18]. This could be attributed to the low lipid content in AV, hence a lack of a gas barrier and hydrophobic properties, and high content of proteins, vitamins, and minerals in AV, which acts as an ideal media for microbial growth.

The addition of lipids to *Aloe* gel is reported to increase the hydrophobic properties and enhance the barrier efficacy of the AV composite coatings. Furthermore, natural antimicrobial agents such as essential oils from various sources have been incorporated in the coatings and films to improve their antimicrobial properties [22,23]. Essential oil from various plant sources serve as natural antimicrobials, and they are classified as generally recognized as safe (GRAS) [24,25]. Among the possible sources, the oil that is extracted from rosehip seeds could be a good alternative as it is rich in unsaturated fatty acids, mainly oleic, linoleic, and linolenic acids [26]. In addition, rosehip oil (RO) has high vitamin C content, minerals, carotenoids, tocopherols, phytosterols, flavonoids, tannins, pectin, sugars, organic acids, and amino acids [27,28]. For this reason, RO is becoming very popular in the cosmetic, pharmaceutic, and food industries [29]. The antimicrobial and antifungal properties of cinnamon oil (CO) have also drawn great attention from many researchers [30,31]. The essential oil of cinnamon has been reported to inhibit the growth of *Fusarium moniliformae* [32] and reported to be effective against *Colletotrichum coccodes*, *Botrytis cinerea*, *Cladosporium herbarum*, *Rhizopus stolonifera*, and *Aspergillus niger* in vitro at 25 and 500 ppm [30].

To the best of our knowledge, there are no studies on the application of AV gel with RO and CO on ‘Bhagwa’ pomegranate arils to preserve the post-harvest quality and extend the shelf life. Therefore, the present investigation is designed to evaluate the impact of RO- and C-based AV gel coatings on the physiochemical attributes, antioxidants, color, and microbial load of pomegranate arils cv. ‘Bhagwa’ during 15 d of storage (5 ± 1 °C, 95 ± 2% RH).

## 2. Materials and Methods

### 2.1. Materials

Pomegranate (*Punica granatum* L. cv. ‘Bhagwa’) fruits were harvested at commercial maturity (TSS 11.0 ± 0.50%, pH 4.2 ± 0.17, % citric acid 2.3 ± 0.15) from a commercial orchard in New Delhi, India. Fresh, regular shaped, uniform sized, healthy fruit (without defect) were phytosanitized by washing in commercial pomegranate fungicide (Teacher™ solution at 600 ppm) for 3 min and allowing the fruit to dry at room temperature. The fruit were immediately stored in a cold room at 5 ± 1 °C before the experiment.

Food grade AV, RO (from *Rosa canina*), and CO (from *Cinnamomum verum)* were procured from Moksha Lifestyle Products Company (New Delhi, India). Black polypropylene terephthalate (PET) punnet sample containers (14.5 × 19.0 × 4 cm) and low-density polyethylene (LDPE) film were purchased from Friends Enterprises (New Delhi, India). The thickness of the PET punnets and LDPE films was 1.52 ± 0.03 mm and 49.915 ± 0.05 µm, respectively. Water vapor, O_2_, and CO_2_ transmission rates of the PET punnets were 27.2 g m^−2^ day^−1^, 60 ± 5 cm^−3^ m^−2^, and 25 ± 3 cm^−3^ m^−2^, respectively. While the water vapor, O_2_, and CO_2_ transmission rates of the LDPE film were 18.25 g m^−2^ day^−1^, 31410 ± 1050 cm^−3^ m^−2^, and 8505 ± 510 cm^−3^ m^−2^, respectively.

### 2.2. Chemicals and Solvents

Culture media (plate count agar, yeast and mold agar, violet red bile agar), methanol (HPLC grade), 2,2-diphenil1-picrylhydrazil (DPPH), Folin–Ciocalteu (FC) reagent, metaphosphoric acid, 2,6-dichlorophenolindophenol, and Tween-80 were purchased from SRL Pvt. Ltd. (New Delhi, India). Cyanidin 3-glucoside was procured from Sigma-Aldrich (New Delhi, India).

### 2.3. Sample Preparation

The pomegranate fruits, plastic containers, knives, and utensils were sterilized with 70% ethanol followed by sodium hypochlorite washing. After washing, the fruit rind was carefully cut at the equatorial zone, and the arils were manually extracted in a sterilized laminar flow (Maxisafe-2030i, Thermo Scientific, Mumbai, India) to reduce the contamination. The arils were collected in the plastic crates and washed with sterile water for 5 min and the excess water was drained from the arils with sterilized paper. The samples were prepared at room temperature (20 ± 2 °C).

### 2.4. Design of Experiment and Coating Treatments

The arils were divided into 11 lots with 3 replications. The arils were subjected to the dipping treatments as per the procedure that is described by Martínez-Romero et al. [18] in the solutions of 10 or 20% AV alone or combined with 0.25 or 0.50% RO or CO for 5 min. Preliminary experiments were carried out to select the doses for detailed experiments and based on that 10 or 20% AV concentration and 0.25 or 0.50% RO or CO concentration taken. In total, 11 treatment combinations were obtained, including a control (without coating), AV (10%), AV (10%) + RO (0.25%), AV (10%) + RO (0.50%), AV (10%) + CO (0.25%), AV (10%) + CO (0.50%), AV (20%), AV (20%) + RO (0.25%), AV (20%) + RO (0.50%), AV (20%) + CO (0.25%), and AV (20%) + CO (0.50%). The excess solution was drained using a sterilized steel colander that was spread on the sterilized blotting paper and left to shade dry. The arils were air-dried for 1 h at room temperature (20 ± 2 °C) in laminar flow. The shade-dried 250 g arils were kept into the sterilized polypropylene boxes followed by sealing them with polyethylene film using an L-shape semi-automated machine and passing them through a shrink wrap machine. A label of 3.0 × 1.5 cm^3^ area was placed onto each package. The packaged samples were stored at 5 ± 1 °C and 95 ± 2% RH for 15 d [33].

### 2.5. Physiological Responses

#### 2.5.1. Ethylene and Respiration Rate

The ethylene production was determined using an ethylene analyzer (ETHAN, Bioconservacion, Barcelona, Spain). Ethylene was measured by placing 250 g arils in 0.5 L airtight plastic container for 1 h before measurement. Gas analysis was performed by inserting the needle that was attached to the gas analyzer through the lid of the container in triplicates for each treatment. The device was auto-calibrated with the atmospheric gas composition. The ethylene production rate was expressed as nmol kg^−1^ h^−1^.

The respiration rate was determined as described by Maurya et al. [34] with slight modifications. The arils were weighed using a weighing balance (Citizen, Mumbai, India) and incubated for 2 h in a 1 L jar. The released CO_2_ by the arils was measured using a CO_2_/O_2_ analyzer (PBI Dansensor CheckMate II Headspace Gas Analyzer, Skandeborg, Danmark) and the results were expressed in CO_2_ nmol kg^−1^ s^−1^.

#### 2.5.2. Weight Loss

Weight loss of the arils was determined according to the method that was described by Kawhena et al. [6]. The weight of the arils was weighed before the experiment and during each sampling day using a digital balance (BSA224S-CW, Sartorius, Goettingen, Germany), and the results were expressed as a percentage:

$$\mathrm{WL}\;\left(\%\right)=\frac{W_{i}-W_{f}}{W_{i}}\times 100$$

where:

*W_i_* = initial weight of the ails
*W_f_* = final weight at the interval of 3 d.

### 2.6. Physical Attributes

#### 2.6.1. Color

The color of the arils was determined in CIE LAB coordinates (L*, a* and b*) using a colorimeter (Minolta Chroma Meter, CR-400, Tokyo, Japan) after calibrating against a white tile background (Illuminants C: Y = 93.6, x = 0.3133, y = 0.3195). The color parameters chroma (C* = (a*^2^ + b*^2^)^1/2^) and hue angle [h^0^ = arctan (b*/a*)] were calculated [35,36]. The mean of 10 measurements was calculated for each replication per treatment. There were three punnets (250 g of arils each) per treatment and the control that were evaluated, and the results were expressed as a mean ± S.E. per interval.

#### 2.6.2. Firmness

The firmness of the arils was measured using a texture analyzer with Warner-Bratzler blade (TAHDI, Stable Micro Systems, Goldalming, UK) with a 35 mm diameter cylindrical probe using Texture Expert Software (Stable Micro Systems, Goldalming, UK) at test speed of 0.05 cm s^−1^ while keeping the pre-and post-test speeds at 0.2 cm s^−1^ [35]. A total of 20 arils were measured per treatment per replication, and the results were expressed as the mean ± S.E. per interval.

### 2.7. Chemical and Enzymatic Attributes

#### 2.7.1. Titratable Acidity, pH and Total Soluble Solids

Titratable acidity (TA) was determined by the potentiometric titration method that was described by Caleb et al. [7]. The extracted juice (1 mL juice diluted in 25 mL distilled water) was titrated against 0.1 N NaOH up to the endpoint of pH 8.2 while using citric acid as a reference, and the results were expressed as g equivalent of citric acid kg^−1^ (g CAE kg^−1^). The arils (10 g) of each sample were juiced separately using a hand juice extractor, and the juice was directly used for pH measurement using a pH meter (Lab India, Mumbai, India) which was already calibrated with pH 4.0 and 7.0 buffer at room temperature (20 ± 3 °C). The total soluble solids (TSS) of the juice were determined by a refractometer (Atago RX-7000i, Tokyo, Japan) with reference to distilled water and the results were expressed in percentage.

#### 2.7.2. L-Phenylalanine Ammonia Lyase (PAL)

The PAL activity was determined according to the method that was described by Qin et al. [37] with slight changes. The aril extract (500 μL) was mixed with 500 μL of 20 mM L-phenylalanine and 2 mL of 50 mM borate buffer (pH 8.8) for 60 min. HCl (6 N) was used in 100 μL volume to stop the reaction, and results were expressed in nmol cinnamic acid h^−1^ mg^−1^ of protein (nmol CA h^−1^ mg^−1^ protein).

### 2.8. Phytochemical Content

#### 2.8.1. Total Phenolic Content (TPC)

The TPC was measured according to the FC method [34]. Diluted aqueous methanolic extract (500 µL) was taken in the test tube, followed by the addition of 450 µL of 50% methanol and 500 µL of the FC reagent. After 2 min, 2.5 mL of sodium carbonate solution (20%) was added to the mixture. Then, the tubes were vortexed and incubated in a dark chamber for 40 min at room temperature. The absorbance of the samples was measured at 725 nm using a UV-Vis spectrophotometer (Shimadzu, UV-2600, Kyoto, Japan). Gallic acid was used as a standard, and the results were expressed as gallic acid equivalent kg^−1^ (g GAE kg^−1^).

#### 2.8.2. Total Anthocyanin Content (TAC)

The TAC was measured by the pH-differential method [38]. The juice (1 mL) was diluted with 9 mL of two buffers (potassium chloride pH 1.0, 0.025 M and sodium acetate pH 4.5, 0.4 M), and the absorbance was measured at 520 nm (potassium chloride) and 700 nm (sodium acetate) using a UV-Vis spectrophotometer (Shimadzu, UV-2600, Kyoto, Japan). The results were expressed as g cyanidin 3-glucoside equivalent kg^−1^ (g CGE kg^−1^).

#### 2.8.3. Ascorbic Acid Content (AAC)

Ascorbic acid (AA) was determined by the 2,6-dichlorophenolindophenol method [34] with some modifications. The juice (1 mL) was diluted with 1% metaphosphoric acid (MPA) (10-fold of 1% MPA) and vortexed for 30 s followed by sonication (32 kHz ultrasound at powers (60 W L^−1^) in an ice bath (8510, Branson Ultrasonics, Mumbai, India) for 5 min. Then, the samples were centrifuged at 40,000× *g* for 20 min at 4 °C using a refrigerated centrifuge (3-18KS, Sigma, Hossein Shakeri, Berlin, Germany), and the supernatant was transferred into a clean tube without disturbing the sediments. The supernatant (1 mL) was diluted with 9 mL of 2,6-dichlorophenolindophenol dye, shaken to mix thoroughly, and incubated for 20 min in the dark. The absorbance of the samples was measured at 515 nm against a blank (1% metaphosphoric acid) using a UV-Vis spectrophotometer (Shimadzu, UV-2600, Kyoto, Japan). The AA content was calculated using a calibration curve of L-ascorbic acid as a standard (0.01–0.1 mg mL^−1^). The results were expressed in g ascorbic acid equivalent kg^−1^ (mg AAE kg^−1^).

### 2.9. Antioxidant Content

The determination of free radical scavenging activity (RSA) (through DPPH*) was determined spectrophotometrically according to the procedure that was described by Maurya et al. [35] with some modifications. Juice extract (150 µL) was diluted in 750 µL of methanol (absolute) and added to 750 µL of 0.1 mM methanolic DPPH* reagent followed by incubation in the dark for 30 min at room temperature. The absorbance was taken spectrophotometrically (Shimadzu 2600, Tokyo, Japan) at 515 nm against methanol and DPPH as a blank and control, respectively. The free RSA (DPPH activity) was calculated using the given formula, and the results were represented in ascorbic acid equivalents (mol AAE kg^−1^).

$$\mathrm{Antioxidant}\;\mathrm{activity}\;\left(\%\right)=1-\frac{\mathrm{Absorbance}\;\mathrm{of}\;\mathrm{sample}}{\mathrm{Absorbance}\;\mathrm{of}\;\mathrm{control}}\times 100$$

### 2.10. Microbial Activity

The microbiological load of the samples was evaluated for aerobic-mesophilic bacteria count, yeast, and mold count, and the coliforms count using plate count agar, yeast mold chloramphenicol agar (YMCA), and violet red bile agar (VRBA), respectively, as described by Standard [39]. The microbial analysis was carried out at 3 d intervals up to 15 d. Pomegranate juice (10 mL) was homogenized in 90 mL of sterile peptone water (physiological solution) using a stomacher (i Mix^®^, Interlab, Paris, France) followed by 10-fold dilutions with peptone water (1.0 mL sample and 9.0 mL peptone water). In order to enumerate microbial load, 1.0 mL of each dilution was poured into a separate Petri dish containing a suitable growth medium. After inoculation, the Petri dishes were incubated at 37 °C for 2 d for bacteria and at 25 °C for 3–5 d for yeast and molds. The results were presented as Log CFU g^−1^.

### 2.11. Sensory Evaluation

The sensory characteristics of the arils were evaluated by a panel of staff, students, and faculties of the National Institute of Food Technology Entrepreneurship and Management (NIFTEM), Kundli, Haryana, India, between the age of 17 and 55 (female:male = 1:1, *n* = 60) for the quality parameters, such as color, flavor, body, texture, and purchase acceptability. The evaluation was done on a 5-point hedonic scale (5 to 1) with 5 = like extremely (very-characteristic of the fruit), 4 = like moderately, 3 = neither like nor dislike (limit of acceptance for consumers), 2 = dislike moderately, and 1 = dislike extremely (non-characteristic of the fruit) [20]. The samples were served randomly as blind labelled with random three-digit codes. The analysis was performed in a cabin with illuminating light, and the potable water was provided for palate cleansing.

### 2.12. Statistical Analysis

All the measurements were done in a completely random design (CRD) with 3 replicates for different parameters and represented as the mean ± SD. Two-way analysis of variance (ANOVA) with Duncan’s multiple comparison post hoc test were used to obtain the significant differences between the data for each storage day using IBM^®^ SPSS (IBM Corp., Armonk, New York, USA) statistics (version 20). The graphs were drawn with the help of GraphPad Prism 5 software. Principle component analysis (PCA) was also applied to explore the connection between the subjected coatings and the quality attributes of the pomegranate arils. The physiochemical attributes of the stored coated arils were evaluated by XL STAT software (www.XLSTAT.com, accessed on 20 June 2022).

## 3. Results and Discussion

### 3.1. Physiological Responses

#### 3.1.1. Ethylene and Respiration Rate

**Figure 1:**
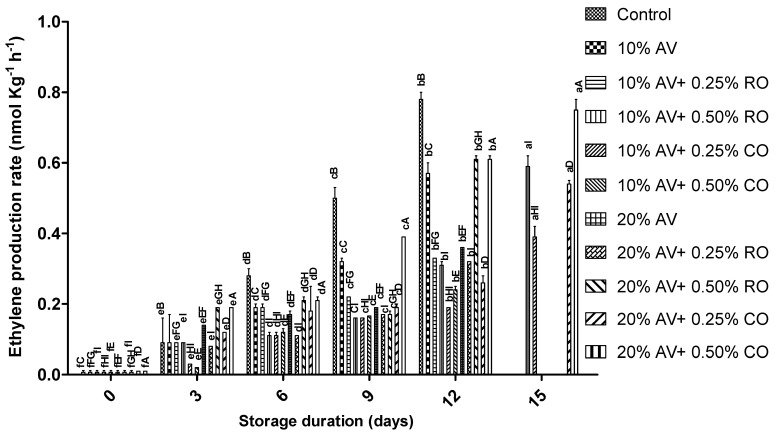
Ethylene production rate (nmol kg^−1^ h^−1^) of ‘Bhagwa’ pomegranate arils that were coated with *Aloe vera* (AV) gel coatings that were enriched with rosehip oil (RO) (0.25% or 0.50%) and cinnamon oil (CO) (0.25% or 0.50%) and stored at 5 ± 1 °C (95 ± 2% RH) for 15 days. The vertical bars represent the standard deviation (SD) of mean values of three replicates (1 punnet = 1 replicate, *n* = 3). The values bearing at least one common small superscript (a, b,…, etc.) and at least one common capital superscript (A, B,…, etc.) letters differ significantly between the days and between the coating solution/concentrations, respectively, at *p* < 0.05 in Duncan’s multiple comparison post hoc test.

The arils’ ethylene production rate increased significantly with the storage duration (Figure 1). The highest increase was found in the control group, while the lowest was observed in 10% AV + 0.25% CO on 12 d of storage. At the end of the experiment (15 d), only four treatments (10% AV + 0.25% CO, 10% AV + 0.50% CO, 20% AV + 0.25% CO, and 20% AV + 0.50% CO) remained. Except for 0 day, all the treatments significantly (*p* ≤ 0.05) inhibited the ethylene production of arils during storage, with a higher inhibition observed in the arils that were treated with AV combined with CO. This could be due to the ability of AV and essential oil to form a barrier against the gas and water, thereby influencing the physiological and physicochemical properties of the treated samples [40]. The ethylene production of the arils that were treated with AV alone or combined with RO or CO corroborates the previous report on pomegranates [41,42].

**Figure 2:**
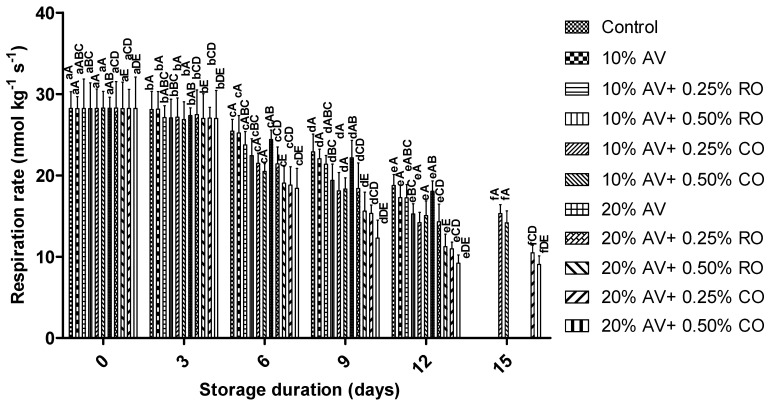
Respiration rate (nmol kg^−1^ s^−1^) of ‘Bhagwa’ pomegranate arils that were coated with *Aloe vera* (AV) gel coatings that were enriched with rosehip oil (RO) (0.25% or 0.50%) and cinnamon oil (CO) (0.25% or 0.50%) and stored at 5 ± 1 °C (95 ± 2% RH) for 15 days. The vertical bars represent the standard deviation (SD) of the mean values of three replicates (1 punnet = 1 replicate, *n* = 3). Values bearing at least one common small superscript (a, b,…, etc.) and at least one common capital superscript (A, B,…, etc.) letters differ significantly between the days and between the coating solution/concentrations, respectively, at *p* < 0.05 in Duncan’s multiple comparison post hoc test.

The respiration rate reduced significantly over the storage period (Figure 2). The control arils showed the highest respiration rate; whereas the respiration rate was reduced in the arils that were treated with 10% AV + 0.25% CO on 12 d of storage. At the end of the storage (15 d), the respiration rate of the treated samples (10% AV + 0.25% CO, 10% AV + 0.50% CO, 20% AV + 0.25% CO, and 20% AV + 0.50% CO) ranged from 9.04 ± 1.06 nmol kg^−1^ s^−1^ to 15.28 ± 1.10 nmol kg^−1^ s^−1^. It can be seen that no significant difference was observed on the 0th day of the storage; while from 0 day onwards, the rate of respiration was found to be reduced significantly (*p* ≤ 0.05) among all the treatments. Again, the inhibitory effects on gas exchange and respiration rates by AV gel coatings can be implicated in this observation. For example, Yousuf and Srivastav [25] observed lower CO_2_ production for the arils that were coated with flaxseed gum (0.3 and 0.6%) that was enriched with lemongrass oil (0–800 ppm) when compared to the uncoated arils, and the authors attributed it to the inhibitory effects of the flaxseed gum on the respiration rate of all the coated arils. Similarly, lower respiration rates were reported when AV gel coating with antimicrobial properties and starch was applied on the pomegranate arils [18,43] and in ‘Acco’ pomegranate arils that were coated with methyl cellulose and gum arabic that was enriched with thyme oil [6].

#### 3.1.2. Weight Loss

**Figure 3:**
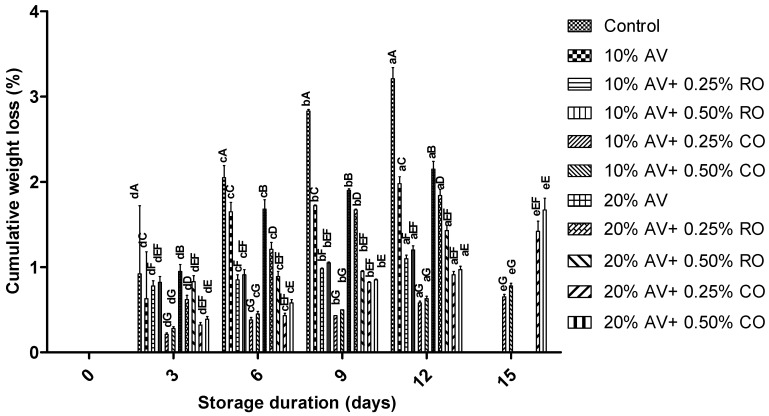
Cumulative weight loss (%) in ‘Bhagwa’ pomegranate arils that were coated with *Aloe vera* gel coatings that were enriched with rosehip oil (0.25% or 0.50%) and cinnamon oil (0.25% or 0.50%) and stored at 5 ± 1 °C (95 ± 2% RH) for 15 days. The vertical bars represent the standard deviation (SD) of the mean values of three replicates (1 punnet = 1 replicate, *n* = 3). Values bearing at least one common small superscript (a, b,…, etc.) and at least one common capital superscript (A, B,…, etc.) letters differ significantly between the days and between the coating solution/concentrations, respectively, at *p* < 0.05 in Duncan’s multiple comparison post hoc test.

Figure 3 indicates a significant (*p* ≤ 0.05) increase in the weight loss of all the samples on 12 d of storage, with a weight loss of 0.51 ± 0.01% observed in the 10% AV + 0.25% CO and 3.16 ± 0.09% was observed in the control. At the end of the experiment (15 d), the weight loss further increased in all the treatments. The observed weight loss in the AV-coated arils in this study was lower than that which was noted in the previous study, wherein the arils were coated with 60, 125, and 250 mL L^−1^ AV gel coating [44]. The lower weight loss in the treated samples could be attributed to the ability of the coatings to form a barrier to water diffusion between the environment and the arils, thus limiting moisture loss and delaying tissue senescence [45]. This notion was reported by Hasheminejad and Khodaiyan [46], who observed decreased weight loss in arils that were treated with clove essential oil + chitosan and stored at 5 °C for 54 d. The results in this study are in agreement with the weight loss magnitude that was recorded in pomegranate arils that were coated with starch + glycerol or starch + glycerol + oil (*N. sativa*) [43,47]. Although the application of coatings on arils maintained weight loss either below or within the acceptable limit for minimally processed fruit for the storage period of 15 d [48]; AV that was combined with CO was more effective than that of AV that was combined with RO in delaying weight loss.

### 3.2. Physical Attributes

#### 3.2.1. Color

A reduction in *h*° was noticed in arils that were treated with AV alone or in combination with oil (CO or RO), but the reduction was more prominent when the concentration of AV and oil increased (Table 1). Further, the reduction in the hue angle was more pronounced during the storage, with the highest value observed in 10% AV + 0.25% CO and the lowest in 20% AV + 0.50% CO at 12 d. A similar observation was reported in a previous study on pomegranate that was coated with different coatings [18,40]. In contrast, the control group had a higher and increasing hue angle with storage time.

The C* value for the control sample decreased during storage from 16.91 ± 0.71 (0 d) to 7.28 ± 0.50 (12 d). The C* values of arils that were treated with AV alone or in combination with oil (RO and CO) varied significantly (*p* ≤ 0.05), with the highest C* value recorded in 10% AV + 0.25% CO coatings and lowest in 20% AV + 0.50% CO on 15 d (Table 1). This observation agrees with previous studies, where the C* value declined in arils that were coated with AV alone or in combination with glycerol or oil, or both [9,40]. Furthermore, 10% AV + 0.25% CO treatment was the most effective in retaining both the color parameters (chroma and hue angle) (Table 1).

#### 3.2.2. Firmness

**Figure 4:**
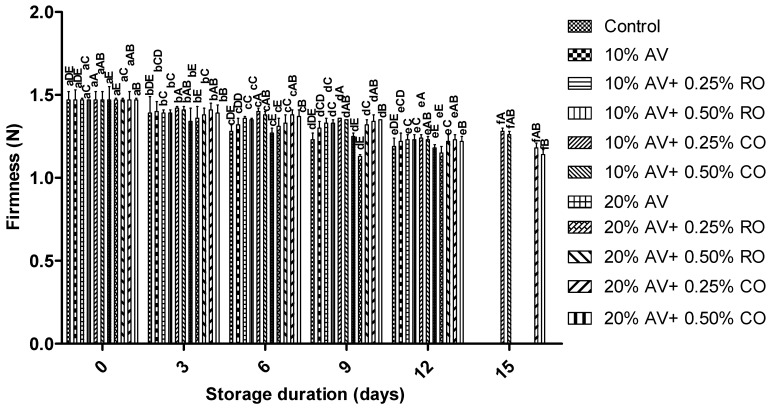
Firmness (N) of ‘Bhagwa’ pomegranate arils that were coated with *Aloe vera* (AV) gel coatings that were enriched with rosehip oil (RO) (0.25% or 0.50%) and cinnamon oil (CO) (0.25% or 0.50%) and stored at 5 ± 1 °C (95 ± 2% RH) for 15 days. The vertical bars represent the standard deviation (SD) of the mean values of three replicates (1 punnet = 1 replicate, *n* = 3). Values bearing at least one common small superscript (a, b,…, etc.) and at least one common capital superscript (A, B,…, etc.) letters differ significantly between the days and between the coating solution/concentrations, respectively, at *p* < 0.05 in Duncan’s multiple comparison post hoc test.

Firmness is one of the vital attributes for customer acceptability. Aril firmness declined with increasing storage duration; however, no significant changes were observed between the control and treated samples on day 0, whereas treatment had a significant effect (*p* ≤ 0.05) on the textural property of arils at the end of the experiment. The treated samples retained a significantly higher firmness than the control samples (Figure 4). The minimal reduction in the firmness was reported for the arils that were treated with 10% AV + 0.25% CO (from 1.47 ± 0.05 N to 1.28 ± 0.02 N) up to 15 d of storage; whereas, the highest reduction was recorded in the control sample, i.e., from 1.47 ± 0.01 N to 1.19 ± 0.02 N up to 12 d. The change in the firmness due to water loss was directly correlated to the reduction in turgor pressure and could be related to the resistance of the outer periderm or increased respiration rate [49]. The results confirmed that the coating materials (AV with essential oils) delayed the post-harvest softening of the arils, resulting in firmness retention. Several studies found higher retention of fruit firmness with AV coatings for various fruits, including papaya [15], nectarine [14], sour cherry [19], and pomegranate arils [18]. A similar observation was reported for the arils that weretreated with other polysaccharides or starch coatings during cold storage [46,50].

### 3.3. Chemical and Enzymatic Attributes

#### 3.3.1. Titratable Acidity, pH, and Total Soluble Solids

**Figure 5:**
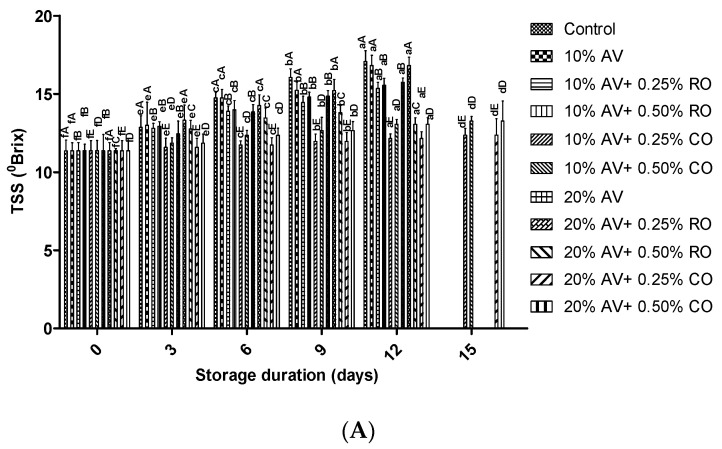

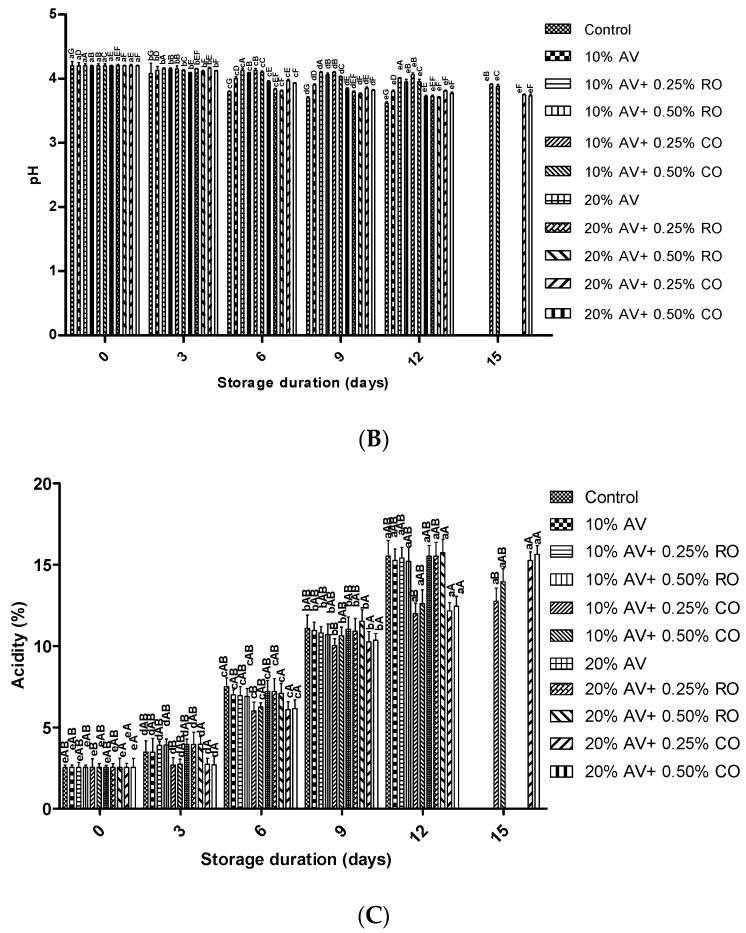
(**A**) TSS (°Brix), (**B**) pH, and (**C**) Titratable acidity (% citric acid equivalent) of ‘Bhagwa’ pomegranate arils that were coated with *Aloe vera* (AV) gel coatings that were enriched with rosehip oil (RO) (0.25% or 0.50%) and cinnamon oil (CO) (0.25% or 0.50%) and stored at 5 ± 1 °C (95 ± 2% RH) for 15 days. The vertical bars represent the standard deviation (SD) of the mean values of three replicates (1 punnet = 1 replicate, *n* = 3). Values bearing at least one common small superscript (a, b,…, etc.) and at least one common capital superscript (A, B,…, etc.) letters differ significantly between the days and between the coating solution/concentrations, respectively, at *p* < 0.05 in Duncan’s multiple comparison post hoc test.

The TSS increased significantly, i.e., the control had the highest (17.07 ± 0.7 °Brix) and the lowest was found in 10% AV + 0.25% CO treated arils (12.36 ± 0.43 °Brix) during the storage (Figure 5A). A significant difference was obtained (*p* ≤ 0.05) between the samples on 3rd, 6th, 9th, 12th, and 15th days of storage. These observations correspond to the TSS values that were recorded for cv. ‘Bhagwa’ pomegranate arils that were coated with 40% and 50% AV gel [47]. This positive effect was due to the oil’s barrier properties against the diffusion of water between the storage environment and the product, thus preventing the change in TSS [45]. Likewise, a lower increase in the TSS was also reported in the pomegranates under cold storage [51]. In contrast, Nabigol and Asghari [44] recorded higher TSS in the pomegranate arils that were coated with AV (60, 125, or 250 mL L^−1^) than the control ones. It was observed that the incorporation of CO in AV is more effective in maintaining the TSS (Figure 5A). Coating with 10% AV + 0.25% CO was the most effective in maintaining the TSS. Pomegranate arils that were coated with starch + glycerol or starch + glycerol + oil (600 ppm seed oil of *N. sativa*) [43] or AV + oil (600 ppm seed oil of *N. sativa*) [47] also supported the present results.

The pH of the freshly extracted aril juice was 4.2, which further decreased with a higher rate in the control sample than in the treated arils. The lowest pH was reported in the control (3.62 ± 0.06) and the highest in the 10% AV + 0.25% CO-treated arils (3.91 ± 0.01) (Figure 5B). There was a significant (*p* ≤ 0.05) difference between the samples on 0 day onwards of storage. On the final day (15 d) of storage, the decreased pH level was between 3.7 and 3.9 for the treated arils. It is also evident that the coating treatments retained the pH longer than the control (Figure 5B). The present values were inconsistent with the results of Hasheminejad and Khodaiyan [46] in chitosan that was enriched with cinnamon oil-coated pomegranate arils.

Titrable acidity at harvest (2.54 ± 0.21 g CAE kg^−1^) increased significantly (*p* ≤ 0.05) with a two-fold increase at each interval, i.e., 0, 3, 6, 9, and 12 d of storage. At the end of the experiment (15 d), the TA was between 12.75 g CAE kg^−1^ (lowest) and 15.63 g CAE kg^−1^ (highest) for 10 AV + 0.25 CO/RO and 10% AV samples, respectively (Figure 5C). These results are in congruence with a previous report where the TA increased in coated pomegranate arils (AV 100% + ascorbic acid 1%) after 8 d of storage at 3 °C [9]. It confirmed that incorporating essential oil in AV was an effective way to maintain the TA. A similar observation was also noted in pomegranate arils that were coated with starch + glycerol or starch + glycerol + oil (600 ppm seed oil of *N. sativa*) [43] or AV + oil (600 ppm seed oil of *N. sativa*) [47]. Moreover, Zarbakhsh et al. [40] also obtained similar results for treated arils under 5 °C storage for 15 d, supporting the present study.

#### 3.3.2. Phenylalanine Ammonia Lyase

**Figure 6:**
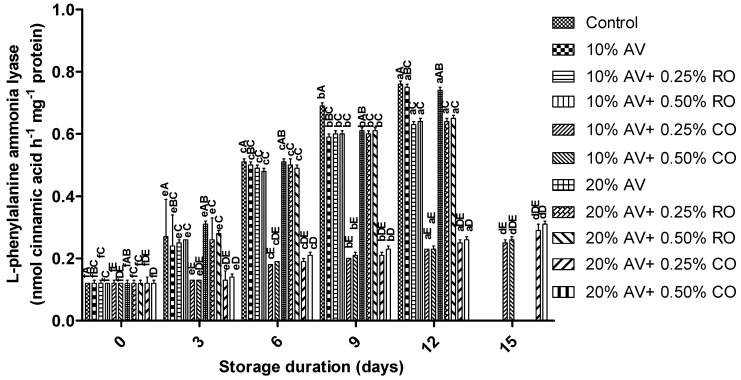
L-phenylalanine ammonia lyase (nmol cinnamic acid h^−1^ mg^−1^ protein) of ‘Bhagwa’ pomegranate arils that were coated with *Aloe vera* (AV) gel coatings that were enriched with rosehip oil (RO) (0.25% or 0.50%) and cinnamon oil (CO) (0.25% or 0.50%) and stored at 5 ± 1 °C (95 ± 2% RH) for 15 days. The vertical bars represent the standard deviation (SD) of the mean values of three replicates (1 punnet = 1 replicate, *n* = 3). Values bearing at least one common small superscript (a, b,…, etc.) and at least one common capital superscript (A, B,…, etc.) letters differ significantly between the days and between the coating solution/concentrations, respectively, at *p* < 0.05 in Duncan’s multiple comparison post hoc test.

The increased PAL activity indicates injured (chilling) fruit, which the treatments can reduce. From zero days onwards, the results showed a significant (*p* ≤ 0.05) increase in the PAL in all the samples. The control (0.768 ± 0.018 nmol CA h^−1^ g^−1^ protein) had the highest, and the 10% AV + 0.25% CO-treated arils showed the lowest PAL activity (0.250 ± 0.015 nmol CA h^−1^ g^−1^ protein) on 12 d of storage (Figure 6). At the end of the experiment, the PAL was significantly higher when the concentration of AV and CO increased. The increase in the PAL in the present study was significantly higher than that which was reported in the previous study, where arils were treated with the salicylic acid (0.7, 1.4 or 2.0 mM) [52].

### 3.4. Phytochemical Content

#### 3.4.1. Total Phenolic Content (TPC)

**Figure 7:**
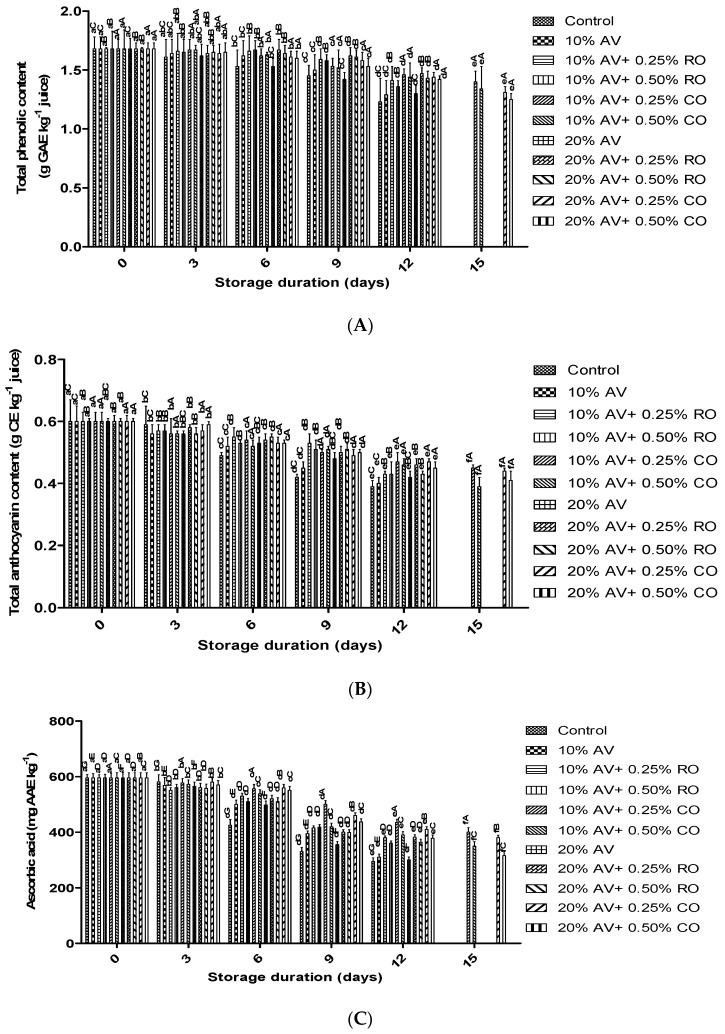
(**A**) Total phenolic content (g GAE kg^−1^ juice), (**B**) total anthocyanin content (g CGE kg^−1^ juice), and (**C**) ascorbic acid (mg AAE kg^−1^) of ‘Bhagwa’ pomegranate arils that were coated with *Aloe vera* (AV) gel coatings that were enriched with rosehip oil (RO) (0.25% or 0.50%) and cinnamon oil (CO) (0.25% or 0.50%) and stored at 5 ± 1 °C (95 ± 2% RH) for 15 days. The vertical bars represent the standard deviation (SD) of the mean values of three replicates (1 punnet = 1 replicate, *n* = 3). Values bearing at least one common small superscript (a, b,…, etc.) and at least one common capital superscript (A, B,…, etc.) letters differ significantly between the days and between the coating solution/concentrations, respectively, at *p* < 0.05 in Duncan’s multiple comparison post hoc test. The vertical bars represent the standard error (SE) of the mean values of three replicates (1 punnet = 1 replicate). The data are expressed in mean SE (*n* = 3). The different letters in the same storage period indicate significant differences at *p* ≤ 0.05.

The highest decrease in TPC was found in the control arils from 1.68 ± 0.01 g GAE kg^−1^ to 1.23 ± 0.02 g GAE kg^−1^ during 12 d of storage. Figure 7A indicates that the TPC of the coated arils was negatively correlated between the AV concentration alone or combined with CO or RO. The arils that were treated with 10% AV + 0.25% CO significantly (*p* ≤ 0.05) retained the level of TPC than the others. The addition of RO and CO controlled the oxidations of phenolics and enzymatic activity, which led to TPC retention in the treated samples. During the post-harvest storage, various studies confirmed the TPC maintenance in the pomegranate arils when they were treated with AV/oil/chitosan materials [53,54]. The higher retention of TPC in the treated arils with AV + CO or RO were supported by previous studies in which the arils were treated with AV alone or in combination with acid [18] or oil [40,43,47]. These observations confirmed that 10% AV + 0.25% CO-treated arils were most effective in retaining the TPC during cold storage.

#### 3.4.2. Total Anthocyanin Content (TAC)

The TAC was decreased gradually during the storage period in all the samples; however, the highest content (0.48 g CE kg^−1^) was observed in 10% AV + 0.25% CO-treated arils on the 12 d (Figure 7B). On the other hand, the control arils had the least amount of TAC (0.39 g CE kg^−1^). Moreover, the treated arils were stored up to 15 d with a moderate level of TAC (0.42 g CE kg^−1^). The previous studies reported a similar trend of declined TAC in the pomegranate arils that were coated with different coatings during storage [4,7,33]. The higher retention of TAC in the AV-treated arils and oil (CO or RO) was reported in previous studies where the arils were treated with AV alone or in combination with oil [43,47].

#### 3.4.3. Ascorbic Acid Content (AAC)

A decrease in the AAC was observed in both the control and the treated samples during the 12 d of storage (Figure 7C). The higher retention of AA was obtained in the AV- along with oil (CO or RO)-treated arils. Similar observations were also seen in the previous studies when arils were treated with AV alone or combined with acid [18,40] or oil [43]. Further, the 10% AV + 0.25% CO coating was the most effective in retaining the vitamin C in the arils. Other coatings, such as starch and glycerol and oil have also been reported to delay vitamin C reduction in the pomegranate arils [43,47].

### 3.5. Antioxidant Content

**Figure 8:**
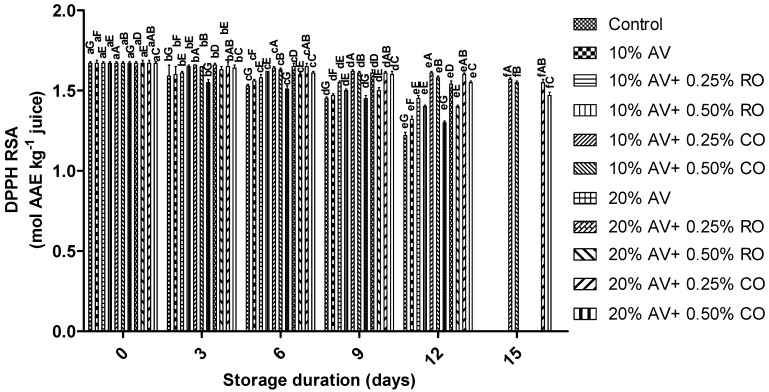
DPPH RSA (mol AAE kg^−1^ juice) of ‘Bhagwa’ pomegranate arils that were coated with *Aloe vera* (AV) gel coatings that were enriched with rosehip oil (RO) (0.25% or 0.50%) and cinnamon oil (CO) (0.25% or 0.50%) and stored at 5 ± 1 °C (95 ± 2% RH) for 15 days. The vertical bars represent the standard deviation (SD) of the mean values of three replicates (1 punnet = 1 replicate, *n* = 3). Values bearing at least one common small superscript (a, b,…, etc.) and at least one common capital superscript (A, B,…, etc.) letters differ significantly between the days and between the coating solution/concentrations, respectively, at *p* < 0.05 in Duncan’s multiple comparison post hoc test.

Initially, no significant difference (*p* ≤ 0.05) was found between the RSA of the control and treated arils, but further, the RSA decreased gradually throughout storage duration. However, the 10% AV + 0.25% CO-treated arils showed the highest RSA than the control (Figure 8). It showed that the coating materials (AV + CO/RO) controlled the loss of phenolics, and it has been confirmed that CO maintains the antioxidant activity of arils for a longer time [55]. A similar declining trend was observed in coated pomegranates with different coatings during cold storage [46,47]. Further, a higher retention of RSA in the arils that were treated AV along with oil (CO or RO) corresponded to the previous report when arils were coated with carboxymethylcellulose + *Zataria multiflora* oil, chitosan + *Zataria multiflora* oil, and AV-coated pomegranate arils [4]. Besides, Zarbakhsh et al. [40] observed higher retention in the antioxidant activity of pomegranate (cv. Jahrom) arils that were treated with citric acid (72.55 ± 3.53%), ascorbic acid (79.69 ± 7.50%), and chitosan (60.58 ± 5.51%) compared to water (62.08 ± 11.38%). The present study showed that the 10% AV + 0.25% CO coating was highly effective in retaining the RSA in treated arils up to 15 d of storage.

### 3.6. Microbial Stability of Arils

Microbial safety is an important parameter for acceptability and higher shelf life of minimally processed products [50]. The influence of different coatings on microbial growth is presented in Table 2. Coating with AV, AV + CO and AV+ RO significantly inhibited bacteria, yeast, and mold growth throughout the storage duration. The control group had the highest growth of microbes from 0.2 to 5 log CFU g^−1^ and survived up to 12 d, while arils that were treated with 20% AV + CO/RO showed the least microbial population up to the end of the experiment (15 d). As per the norms, 5 log CFU g^−1^ and 7 log CFU g^−1^ are the maximum acceptable limits for yeast and mold and total bacterial counts, respectively [56]. The obtained results of treated arils were under the recommended limit and thus safe for consumption. In addition, CO was more effective in inhibiting microbial growth than RO. The antimicrobial activity of edible coatings has been confirmed by previous studies on several fruits and vegetables, including nectarines [57], kiwifruit [12], table grapes [58], and pomegranate arils [18,59]. The bioactive compounds of AV, such as aloe-emodin and aleonin, have been reported to exhibit antifungal activity against *Aspergillus*, *Cladosporium*, and *Fusarium* [60]. Furthermore, the antimicrobial activity of CO and RO has also been reported [61,62].

### 3.7. Sensory Evaluation

**Figure 9:**
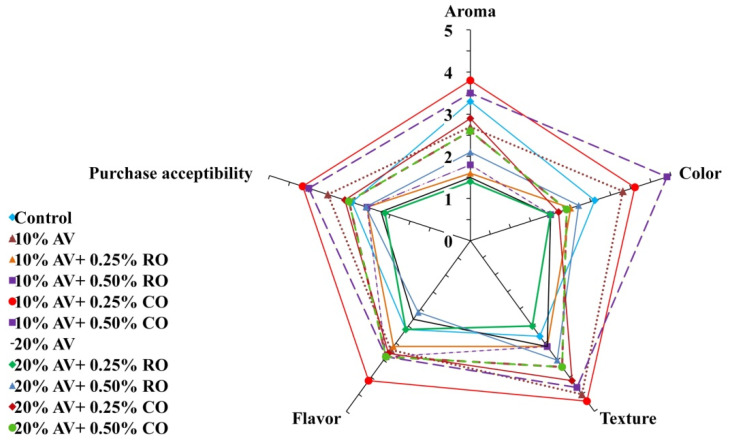
Sensory attributes (flavor, texture, color, aroma, and purchase acceptability) of ‘Bhagwa’ pomegranate arils that were coated with *Aloe vera* (AV) gel coatings that were enriched with rosehip oil (RO) (0.25% or 0.50%) and cinnamon oil (CO) (0.25% or 0.50%) and stored at 5 ± 1 °C (95 ± 2% RH) for 15 days. The data were captured after 9 days of storage during the 15 days trials due to safety precautions. The vertical bars represent the standard deviation (SD) of the mean values of three replicates (1 punnet = 1 replicate, *n* = 3). The data are expressed as the mean ± SD (*n* = 3).

Figure 9 depicts the effects of different coatings on the sensory attributes (aroma, flavor, color, texture, and purchase acceptability) of arils, and the results are presented for data on day 9 of the experiment. The results revealed that the highest score (3–4) was assigned to the 10% AV + 0.25% CO-treated arils followed by 10% AV + 0.25% RO. Moreover, 10% AV + 0.25% CO was regarded as the most suitable coating material for maintaining texture, flavor, aroma, and purchasing acceptability of the arils. The coating material maintained the flavor and texture for a longer time due to the retention of water loss and oxidation. A similar decline in the sensory attributes were reported when pomegranate arils were treated with different coating materials such as chitosan [9], AV alone, or in combination with ascorbic acid [18]. A recent study also concluded that the essential oil coating retained the color, flavor, and texture in arils up to 24 d at 5 °C storage [46].

### 3.8. Principal Component Analysis (PCA)

**Figure 10:**
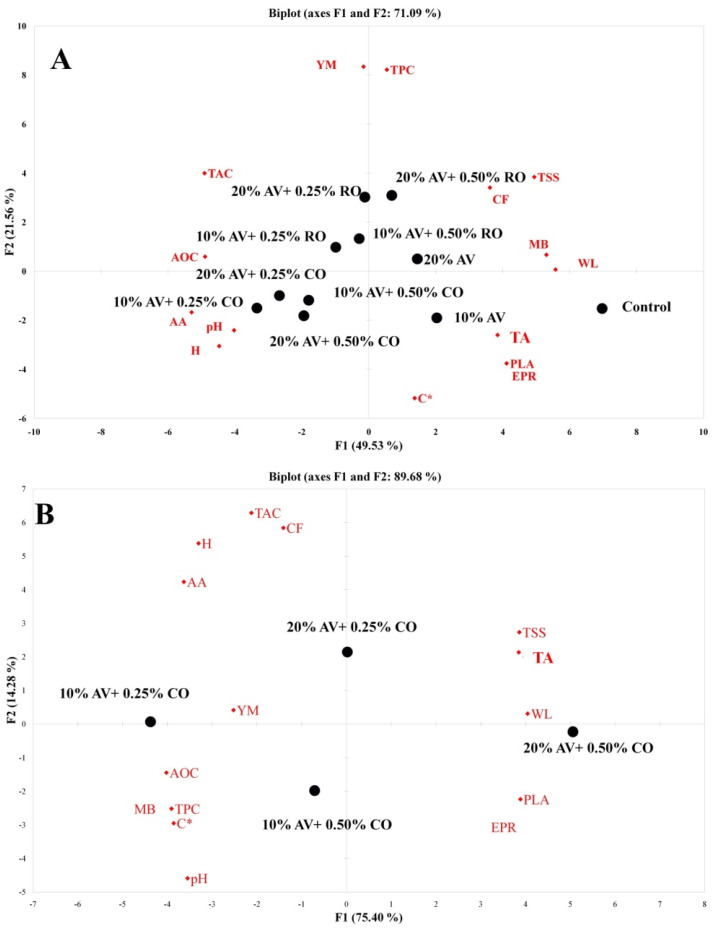
PCA bi-plot of the physiological, physical, chemical, enzymatic, phytochemical, and antioxidant attributes of ‘Bhagwa’ pomegranate arils that were coated with *Aloe vera* (AV) gel coatings that were enriched with rosehip oil (RO) (0.25% or 0.50%) and cinnamon oil (CO) (0.25% or 0.50%) and stored at 5 ± 1 °C (95 ± 2% RH) for 15 days. Where two principal components of bi-plots were (**A**) 48.53% and 21.56% for 9 day pomegranate arils and (**B**) 75.40% and 14.28% for 15 day pomegranate arils.

A PCA is normally conducted to fetch the correlation between the quality attributes and the grouping of the samples (Figure 10) (A: 9 d and B: 15 d). A PCA was conducted to find the relationship between the applied treatments (coating materials) and various quality attributes of the arils, which decide its quality and shelf life. The PCA is composed of two principal components having different data variability for differently treated samples. These two principal components varied for arils among the treatments, i.e., 48.53% and 21.56% for 9 d and 75.40% and 14.28% for 15 d. The AA, firmness, respiration rate, and pH were correlated with 10% AV + 0.25% CO (Figure 10A). The acidity, PAL, ethylene production rate, and C* were correlated with 10% AV, and weight loss was correlated with the control. TPC and TSS were correlated with 20% AV + 0.50% RO, while TAC and RSA were positively correlated with 20% AV + 0.25% RO. Figure 10B shows the positive correlation between RSA, firmness, respiration rate, TPC, C*, and pH with 10% AV + 0.25% CO. Additionally, PAL, weight loss, TA, and TSS were correlated with 20% AV + 0.50% CO. PCA analysis also supports that the AV + CO coating combinations were more effective in retaining the quality attributes and shelf life of pomegranate arils.

## 4. Conclusions

The present study suggested that applying different treatments significantly influences the quality attributes of pomegranate arils. The AV + RO coating limited ethylene production more than the other coating combinations, while TPC was more retained in the AV + CO-coated arils. The AV + CO coating extended the shelf life of the arils for up to 15 d compared to 12 d for other coating combinations. Furthermore, the microbial load was significantly (*p* ≤ 0.05) reduced in arils that were coated with AV + CO followed by (AV + RO) and AV coating materials, suggesting microbial safety of the treated arils. In addition to higher retention of TPC, TAC, RSA, and ascorbic acid content, the coating materials also affected the organoleptic acceptability. Principal component analysis confirmed AV + CO as the most effective coating combination.

## Figures and Tables

**Table 1:** The effects of *Aloe vera* (AV) gel coating that was enriched with rosehip oil (RO) (0.25% or 0.50%) and cinnamon oil (CO) (0.25% or 0.50%) on the color attributes that are reported as the hue angle (*h*°) and chroma (C*) of ‘Bhagwa’ pomegranate arils during storage at 5 ± 1 °C (95 ± 2% RH) for 15 days.

Color Attributes	Storage Duration (Days)						
	Treatment	0	3	6	9	12	15
*h*°	Control	23.91 ± 0.95 ^aA^	24.65 ± 0.67 ^bA^	24.01 ± 0.56 ^cA^	24.98 ± 0.58 ^dA^	29.01 ± 0.95 ^eA^	ND
	10% AV	20.76 ± 1.25 ^aEF^	15.43 ± 0.95 ^bEF^	15.13± 1.15 ^cEF^	14.09 ± 1.20 ^dEF^	10.84 ± 1.21 ^eEF^	ND
	10% AV + 0.25% RO	20.72 ± 0.65 ^aD^	18.97 ± 0.85 ^bD^	16.31 ± 0.95 ^cD^	14.86 ± 1.25 ^dD^	13.01 ± 1.05 ^eD^	ND
	10% AV + 0.50% RO	21.21 ± 0.95 ^aDE^	16.04 ± 0.65 ^bDE^	15.65 ± 0.55 ^cDE^	15.06 ± 0.35 ^dDE^	12.61 ± 0.75 ^eDE^	ND
	10% AV + 0.25% CO	20.68 ± 1.28 ^aB^	20.03 ± 1.34 ^bB^	19.15 ± 1.45 ^cB^	18.81 ± 1.23 ^dB^	17.02 ± 1.20 ^eB^	16.65 ± 1.10 ^fB^
	10% AV + 0.50% CO	20.71 ± 1.32 ^aC^	19.56 ± 1.45 ^bC^	18.6 ± 0.96 ^cC^	17.46 ± 0.65 ^dC^	15.74 ± 0.75 ^eC^	16.65 ± 1.10 ^fC^
	20% AV	20.14 ± 0.75 ^aGH^	15.05 ± 0.85 ^bGH^	14.61 ± 0.53 ^cGH^	13.42 ± 0.54 ^dGH^	9.54 ± 0.58 ^eGH^	ND
	20% AV + 0.25% RO	20.69 ± 1.28 ^aFG^	17.21 ± 1.30 ^bFG^	15.24 ± 0.95 ^cGH^	12.78 ± 0.45 ^dFG^	10.13 ± 0.25 ^eFG^	ND
	20% AV + 0.50% RO	21.08 ± 0.55 ^aH^	16.48 ± 0.38 ^bH^	12.32 ± 0.45 ^cH^	12.04 ± 0.85 ^dH^	9.87 ± 0.55 ^eH^	ND
	20% AV + 0.25% CO	21.12 ± 0.65 ^aB^	20.02 ± 0.65 ^bB^	19.87 ± 0.35 ^cB^	18.98 ± 0.85 ^dB^	19.09 ± 0.71b ^eB^	17.03 ± 0.96 ^fB^
	20% AV + 0.50% CO	21.26 ± 0.75 ^aDE^	17.03 ± 0.73 ^bDE^	15.26 ± 0.95 ^cDE^	14.89 ± 0.74 ^dDE^	14.64 ± 0.73 ^eDE^	17.03 ± 0.96 ^fDE^
C*	Control	16.91 ± 0.71 ^aC^	15.12 ± 2.19 ^bC^	12.06 ± 0.85 ^cC^	10.21 ± 0.82 ^dc^	7.28 ± 0.50 ^eC^	ND
	10% AV	16.56 ± 0.70 ^aAB^	14.44 ± 1.78 ^bAB^	13.01 ± 0.56 ^cAB^	12.03 ± 0.55 ^dAB^	10.16 ± 0.85 ^eAB^	ND
	10% AV + 0.25% RO	16.45 ± 0.67 ^aA^	14.11 ± 0.85 ^bA^	12.97 ± 0.95 ^cA^	12.75 ± 0.65 ^dA^	10.91 ± 0.69 ^eA^	ND
	10% AV + 0.50% RO	16.32 ± 0.53 ^aAB^	14.56 ± 0.84 ^bAB^	13.05 ± 0.58 ^cAB^	11.11 ± 0.82 ^dAB^	10.03 ± 0.75 ^eAB^	ND
	10% AV + 0.25% CO	16.35 ± 0.65 ^aA^	13.96± 0.55 ^bA^	12.97± 0.95 ^cA^	13.01± 0.65 ^dA^	12.59± 0.68 ^eA^	11.85 ± 0.910 ^eA^
	10% AV + 0.50% CO	16.25 ± 0.62 ^aAB^	14.95 ± 0.62 ^bAB^	13.89 ± 0.56 ^cAB^	13.02 ± 0.85 ^dAB^	11.02 ± 0.65 ^eAB^	9.53 ± 0.55 ^eAB^
	20% AV	16.39 ± 0.51 ^aBC^	12.94 ± 0.68 ^bBC^	12.83 ± 0.65 ^cBC^	12.06 ± 0.75 ^dBC^	9.41 ± 0.65 ^eBC^	ND
	20% AV + 0.25% RO	16.35 ± 0.81 ^aAB^	12.93 ± 0.65 ^bAB^	12.61 ± 0.65 ^cAB^	12.98 ± 0.75 ^dAB^	11.32 ± 0.68 ^eAB^	ND
	20% AV + 0.50% RO	16.28 ± 0.60 ^aC^	12.12± 0.76 ^bC^	11.68 ± 0.55 ^cC^	10.94 ± 0.45 ^dC^	10.56 ± 0.58 ^eC^	ND
	20% AV + 0.25% CO	16.30 ± 0.65 ^aA^	14.98 ± 0.55 ^bA^	14.01 ± 0.65 ^cA^	13.72± 0.74 ^dA^	11.85 ± 0.85 ^eA^	10.48 ± 0.54 ^eA^
	20% AV + 0.50% CO	16.20 ± 0.75 ^aAB^	14.93 ± 0.57 ^bAB^	14.64 ± 0.71 ^cAB^	13.52 ± 0.58 ^dAB^	10.02 ± 0.68 ^eAB^	9.32 ± 0.65 ^eAB^

Values expressed as the mean ± SD, *n* = 3. Values bearing at least one common small superscript (a, b,…, etc.) and at least one common capital superscript (A, B,…, etc.) letters differ significantly between the columns (between days) and between the rows (between coating concentrations), respectively, at *p* < 0.05 in Duncan’s multiple comparison post hoc test. ND—not determined due to complete loss/spoilage of samples.

**Table 2:** Effects of *Aloe vera* (AV) gel coating that was enriched with rosehip oil (RO) (0.25% or 0.50%) and cinnamon oil (CO) (0.25% or 0.50%) on the yeast and mold counts (log CFU g^−1^) and total plate count (log CFU g^−1^) of ‘Bhagwa’ pomegranate arils during storage at 5 ± 1 °C (95 ± 2% RH) for 15 days.

Microbial Quality	Storage Duration (Days)						
	Treatment	0	3	6	9	12	15
Total plate count (log cfu g^−1^)	Control	2.1± 1.05 ^cA^	4.0 ± 1.06 ^bA^	4.4 ± 1.12 ^abA^	4.8 ± 1.45 ^abA^	5.0 ± 1.31 ^aA^	ND
	10% AV	1.8 ± 1.11 ^cAB^	3.3 ± 1.61 ^bAB^	3.5 ± 1.54 ^abAB^	4.1 ± 1.74 ^abAB^	4.4 ± 2.01 ^aAB^	ND
	10% AV + 0.25% RO	1.4 ± 1.22 ^cAB^	3.3 ± 1.15 ^bAB^	3.6 ± 2.02 ^abAB^	3.7 ± 1.40 ^abAB^	3.8 ± 1.35 ^aAB^	ND
	10% AV + 0.50% RO	1.5 ± 1.25 ^cAB^	3.1 ± 1.05 ^bAB^	3.7 ± 1.21 ^abAB^	3.6 ± 1.33 ^abAB^	3.9 ± 1.11 ^aAB^	ND
	10% AV + 0.25% CO	2.1 ± 1.38 ^cAB^	3.2 ± 1.61 ^bAB^	3.5 ± 1.13 ^abAB^	3.7 ± 1.21 ^abAB^	3.8 ± 1.15 ^aAB^	4.1± 1.76 ^aAB^
	10% AV + 0.50% CO	2.2 ± 1.20 ^cAB^	3.1 ± 1.15 ^bAB^	3.2 ± 0.85 ^abAB^	3.4 ± 1.15 ^abAB^	3.8 ± 1.41 ^aAB^	4.0 ± 1.76 ^aAB^
	20% AV	2.1 ± 1.35 ^cAB^	3.3 ± 1.23 ^bAB^	3.8 ± 1.05 ^abAB^	4.0 ± 1.35 ^abAB^	4.2 ± 1.08 ^aAB^	ND
	20% AV + 0.25% RO	2.1 ± 1.15 ^cAB^	3.3 ± 2.15 ^bAB^	3.5 ± 1.07 ^abAB^	3.8 ± 1.23 ^abAB^	4.0 ± 1.05 ^aAB^	ND
	20% AV + 0.50% RO	2.1 ± 1.08 ^cAB^	2.7 ± 1.05 ^bAB^	3.4 ± 1.25 ^abAB^	3.9 ± 1.44 ^abAB^	4.0 ± 1.42 ^aAB^	ND
	20% AV + 0.25% CO	2.2 ± 1.05 ^cAB^	2.5 ± 1.44 ^bAB^	2.9 ± 1.35 ^abAB^	3.3 ± 1.14 ^abAB^	3.5 ± 1.14 ^aAB^	3.7 ± 1.83 ^aAB^
	20% AV + 0.50% CO	2.2 ± 2.05 ^cB^	2.3 ± 1.25 ^bB^	2.6 ± 1.05 ^abB^	2.8 ± 1.0 ^abB^	3.3 ± 1.22 ^aB^	3.4 ± 1.36 ^aB^
Yeast and Mold count (log cfu g^−1^)	Control	1.3± 1.15 ^cA^	3.2 ± 1.46 ^bA^	3.5 ± 1.47 ^abA^	3.7 ± 1.05 ^aA^	3.8 ± 1.61 ^aA^	ND
	10% AV	1.3 ± 1.44 ^cA^	2.5 ± 1.11 ^bA^	3.1 ± 1.64 ^abA^	3.2 ± 1.24 ^aA^	4.0 ± 1.31 ^aA^	ND
	10% AV + 0.25% RO	0.1 ± 1.12 ^cA^	3.0 ± 1.45 ^bA^	3.1 ± 2.12 ^abA^	3.3 ± 1.23 ^aA^	3.4 ± 1.11 ^aA^	ND
	10% AV + 0.50% RO	0.1 ± 1.45 ^cA^	2.7 ± 1.15 ^bA^	2.9 ± 1.61 ^abA^	3.2 ± 1.33 ^aA^	3.4 ± 1.31 ^aA^	ND
	10% AV + 0.25% CO	0.1 ± 1.38 ^cA^	3.7 ± 1.51 ^bA^	3.8 ± 1.63 ^abA^	3.0 ± 1.71 ^aA^	3.1 ± 1.25 ^aA^	3.5 ± 1.35 ^aA^
	10% AV + 0.50% CO	1.3 ± 1.40 ^cA^	1.5 ± 1.15 ^bA^	2.7 ± 1.25 ^abA^	3.0 ± 1.65 ^aA^	3.1 ± 1.61 ^aA^	3.4 ± 1.70 ^aA^
	20% AV	1.3 ± 1.11 ^cA^	2.8 ± 1.43 ^bA^	3.2 ± 1.55 ^abA^	3.4 ± 1.22 ^aA^	3.5 ± 1.34 ^aA^	ND
	20% AV + 0.25% RO	0.1 ± 0.21 ^cA^	3.1 ± 1.35 ^bA^	3.2 ± 1.27 ^abA^	3.3 ± 1.13 ^aA^	3.5 ± 1.11 ^aA^	ND
	20% AV + 0.50% RO	0.1 ± 0.18 ^cA^	2.8 ± 1.05 ^bA^	3.2 ± 1.15 ^abA^	3.4 ± 1.04 ^aA^	3.5 ± 1.12 ^aA^	ND
	20% AV + 0.25% CO	1.0 ± 1.11 ^cA^	1.9 ± 1.14 ^bA^	3.0 ± 1.14 ^abA^	3.0 ± 1.24 ^aA^	3.2 ± 1.33 ^aA^	3.1 ± 0.99 ^aA^
	20% AV + 0.50% CO	1.3 ± 1.35 ^cA^	1.4 ± 1.35 ^bA^	2.6 ± 1.75 ^abA^	2.9 ± 1.04 ^aA^	3.0 ± 1.62 ^aA^	3.0 ± 1.66 ^aA^
Coliform count (log cfu g^−1^)	Control	0.0 ± 0.01 ^cA^	2.8 ± 1.56 ^cA^	3.1 ± 1.42 ^bA^	3.3 ± 1.15 ^aA^	3.5 ± 1.21 ^aAB^	ND
	10% AV	0.0 ± 0.02 ^cAB^	0.0 ± 0.05 ^cAB^	2.5 ± 1.12 ^bAB^	3.0 ± 1.74 ^aAB^	3.1 ± 1.31 ^aAB^	ND
	10% AV + 0.25% RO	0.0 ± 0.02 ^cAB^	2.3 ± 1.01 ^cAB^	2.6 ± 1.32 ^bAB^	2.7 ± 1.20 ^aAB^	2.8 ± 1.11 ^aAB^	ND
	10% AV + 0.50% RO	0.0 ± 0.15 ^cA^	2.5 ± 1.35 ^cA^	2.6 ± 1.31 ^bA^	3.0 ± 1.73 ^aA^	3.4 ± 1.61 ^aA^	ND
	10% AV + 0.25% CO	0.0 ± 0.20 ^cA^	0.0 ± 0.01 ^cA^	2.2 ± 1.15 ^bA^	3.6 ± 1.65 ^aA^	3.7 ± 1.12 ^aA^	3.5 ± 1.46 ^aA^
	10% AV + 0.50% CO	0.0 ± 0.50 ^cAB^	0.0 ± 0.08 ^cAB^	0.0 ± 0.07 ^bAB^	3.0 ± 1.51 ^aAB^	3.2 ± 1.61 ^aAB^	3.3 ± 1.21 ^aAB^
	20% AV	0.0 ± 0.06 ^cAB^	0.0 ± 0.06 ^cAB^	2.5 ± 1.12 ^bAB^	2.7 ± 1.13 ^aAB^	3.1 ± 1.07 ^aAB^	ND
	20% AV + 0.25% RO	0.0 ± 0.10 ^cAB^	0.0 ± 0.14 ^cAB^	3.0 ± 0.09 ^bAB^	3.1 ± 1.35 ^aAB^	3.3 ± 1.18 ^aAB^	ND
	20% AV + 0.50% RO	0.0 ± 0.10 ^cAB^	0.0 ± 0.08 ^cAB^	2.8 ± 1.85 ^bAB^	3.0 ± 1.24 ^aAB^	3.1 ± 1.12 ^aAB^	ND
	20% AV + 0.25% CO	0.0 ± 0.11 ^cAB^	0.0 ± 0.08 ^cAB^	1.0 ± 1.25 ^bAB^	2.1 ± 1.05 ^aAB^	3.1 ± 1.41 ^aAB^	3.4 ± 1.03 ^aAB^
	20% AV + 0.50% CO	0.0 ± 0.07 ^cB^	0.0 ± 0.18 ^cB^	0.0 ± 0.40 ^bB^	2.1 ± 1.04 ^aB^	2.4 ± 1.62 ^aB^	3.2 ± 1.56 ^aB^

Values expressed as the mean ± SD, *n* = 3. Values bearing at least one common small superscript (a, b,…, etc.) and at least one common capital superscript (A, B) letters differ significantly between the columns (between days) and between the rows (between Coating concentrations), respectively, at *p* < 0.05 in Duncan’s multiple comparison post hoc test. ND—not determined due to complete loss/spoilage of samples.

## Data Availability

Data will be made available on request.
